# A randomized controlled trial of WeChat-based cognitive behavioral therapy intervention to improve cancer-related symptoms in gynecological cancer survivors: study protocol

**DOI:** 10.1186/s12913-022-08443-y

**Published:** 2022-08-17

**Authors:** Liyuan Sun, Yanling Tao, Shening Zhu, Ke Liu

**Affiliations:** 1 Health Science Center, Shenzhen University, A1-403, 1066 Xueyuan Road, Nanshan District, Shenzhen, 518060 China; 2 Longgang Central Hospital of Shenzhen, Shenzhen, China; 3 Shenzhen Maternity&Child Healthcare Hospital, Southern Medical University, Shenzhen, China

**Keywords:** Oncology, Quality of life, Cognitive behavioral therapy, Randomized controlled trial

## Abstract

**Background:**

Gynecological malignant patients often have significant psychological and physical problems. The feasibility and generalizability of traditional intervention method is low due to the high time and labor cost, large number of gynecological malignant tumor patients in China, as well as shortage of health professionals. Therefore, it is necessary to design an alternative, innovative, and easily accessible intervention method. This study aims to evaluate the effect of WeChat-based intervention on anxiety, depression and disease-related symptoms of patients with gynecological malignant tumors during rehabilitation.

**Methods:**

A single-blinded, randomized, controlled, parallel-group pre-test and repeated post-test design will be conducted. A total of 76 participants will be randomly divided into the intervention group and control group. Anxiety and depression, disease-related symptoms, coping ability, benefit finding and quality of life will be measured at baseline and repeated immediately after the intervention (test 1), 3 months (test 2) and 6 months (test 3) after the intervention.

**Discussion:**

As the first randomized controlled trial with rigorous research design for patients with gynecological malignant tumors in the rehabilitation stage in China, this study will provide evidences for the effectiveness of the WeChat platform during intervention of patients with gynecological malignant tumors in the rehabilitation stage. The results are helpful to further explore the effect of WeChat-based intervention on improving patients' anxiety and depression, disease-related symptoms, and quality of life.

**Trial registration:**

Chinese Clinical Trial Registry: ChiCTR2100053450, Registered 21 November 2021,http://www.chictr.org.cn/searchproj.aspx

## Background

Gynecological malignant tumor is a major disease threatening women's health, and has high incidence and high mortality [1]. There are many types of gynecological malignant tumors, among which ovarian cancer, cervical cancer, and endometrial cancer are the most common [2]. In recent years, the incidence rate has been increasing steadily, and the age of onset has gradually become younger. According to the report released by the International Agency for Research on Cancer (IARC), there were about 1,246,000 new cases of ovarian cancer, cervical cancer, and endometrial cancer and about 584,000 deaths in 2018 alone [1, 2]. The new cases of these three tumors in Chinese women is about 233,000, accounting for one fifth of the global cases, which greatly threatens the health and life of these patients [2, 3].

To treat gynecological malignant tumors, comprehensive treatment is generally needed, such as surgery, chemotherapy, or radiotherapy, which often leads to anatomical changes of the patient's reproductive system, menopause, vaginal dryness, hot flashes, and sexual intercourse pain [3]. The adverse effects result in damage to the reproductive, sexual, and urinary functions. Patients often have significant sexual psychological problems and body image disorders [4]. Moreover, the incidence of anxiety and depression was found to be high among these patients [5, 6]. Currently, the outcome for the treatment of malignant tumors is evaluated by not only the remission of clinical symptoms and the prolongation of survival time, but also the quality of life. Therefore, more attention should be paid to the psychological problems of patients with gynecological malignant tumors in the rehabilitation stage, in the purpose of alleviating symptoms and improving the patients’ quality of life.

A number of existing studies have shown that psychological intervention played a positive role in improving the sexual psychological problems and emotional disorders in patients with gynecological malignant tumors. Proper intervention satisfied the emotional and psychological needs of patients and improved the physical function by alleviating such symptoms as anxiety, depression, fatigue, pain, and insomnia [7–9]. Studies based in China have also confirmed the positive effect of psychological intervention for patients with gynecological malignant tumors [10, 11]. Recently, the cognitive behavior therapy (CBT), as a psychotherapy method in survival nursing, has attracted more and more attention. CBT plays an important role in group psychotherapy. By changing patients' incorrect cognition of the disease and conducting behavioral training, CBT helps the patients to establish reasonable cognition, change poor psychological state and negative coping style, thereby improving disease-related symptoms and the quality of life [12–15]. Many studies have shown that CBT is a beneficial treatment option for a variety of cancer patients [16–18]. Hersch et al. showed that CBT reduced psychological pain, anxiety, and depression [19]. However, current studies on CBT intervention for cancer patients are mainly focused on breast cancer, followed by prostate cancer [20]. Few studies have been carried out on patients with gynecological malignant tumors.

Moreover, traditional CBT interventions are carried out in a face-to-face setting. Studies have pointed out that due to cultural factors, most patients in China were unwilling to discuss sex psychology problems publicly [21, 22]. Stead et al. showed that only about a quarter of medical staff have been talked to about this problem. Thus, in many cases, the special psychological problems of patients with gynecological malignant tumors were not addressed [23]. Instead of face-to-face intervention, gynecological malignant tumor patients are more willing to participate in online support groups, due to the anonymity of discussion, which helped the patients feel comfortable talking about and solve these problems [24]. Further, the feasibility and generalizability of traditional intervention method is low due to the high time and labor cost, large number of gynecological malignant tumor patients in China, as well as shortage of health professionals. Therefore, it is necessary to design an alternative, innovative, and easily accessible intervention method. The development of internet has enable the possibility of convenient and accessible of online, virtual intervention, the Internet-based intervention can be conducted through multiple media formats to customize information, which saves participants' time and cost, and makes it easier to participate. Thus, internet-based intervention is an innovative and convenient intervention method, and has the ability to reach more target patients [25, 26]. Internet cognitive behavioral therapy (ICBT) produced the same results as face-to-face therapy in terms of mental health improvement. ICBT is being increasingly adopted in the study of physical health of patients with chronic diseases [27, 28]. A review by Andersson et al. showed that the effects of ICBT and face-to-face CBT on a series of mental and physical diseases were the same, which provided evidences for the adoption of ICBT [29].

In China, WeChat is the most widely used social network platform. At present, there are 902 million active users every day, and each user spend an average of 66 min on the platform every day [30]. WeChat has many different functions, such as private or group texts or voice messages, video calls or meetings, electronic document transmission, subscription and browsing of public accounts and information publishing [31, 32]. Because WeChat has a variety of interactive functions and a large amount of users, and is convenient, timely, and affordable [32], it has been slowly changing the way people receive information. It breaks the time and space limitations of traditional classrooms, improves autonomous learning, and strengthens cooperation with peers [33]. WeChat has been used for behavioral intervention and disease prevention and control, such as cancer, malaria, asthma, chronic sinusitis, diabetes and weight loss [34–39]. Some studies have used WeChat-based group intervention for malignant tumor patients. The attrition rate of participants was relatively low and the acceptability among users was good. Some patients said that they have found a sense of belonging in the patient group, and have "people and places that can speak their hearts". As a result, both online and offline communication was very active [40]. WeChat can optimize the communication between patients and medical staff [32],and when doctors withdrew from the WeChat group after the group intervention, group members can still maintain interactive communication in the WeChat group. Through the platform, the members spontaneously organized offline collective outings and other activities to provide continuous psychological support between group members [40]. However, there is still no study on the effect of WeChat on patients with gynecological malignant tumors in the rehabilitation stage. Therefore, the objective of the present study is to explore the WeChat-based CBT(WCBT) intervention model. By carrying out psychological intervention and follow-up management of patients with gynecological malignant tumors through WeChat, its impact on the patients’ physical and psychological symptoms will be explored.

## Methods/design

### Aims

The objective of this study was to evaluate the effect of CBT based on the WeChat platform on anxiety, depression, and disease-related symptoms of patients with gynecological malignant tumors in the rehabilitation stage, thereby establishing an intervention model to improve the patient’s coping ability, benefit finding and quality of life. The study hypothesizes that compared with the control group at baseline, test 1, test 2 and test 3, patients in the intervention group will report statistically significant:

1. The degree of anxiety and depression is decreased;
2. Disease-related symptoms are reduced;
3. Coping ability, benefit finding and quality of life are improved.

### Study design

#### Trial design

A single-blinded, randomized, controlled, parallel group pre-test, and repeated post-test design will be conducted to investigate the effect of WeChat-based Cognitive behavioral therapy for Chinese gynecological cancer survivors.

#### SPIRIT statement

**Fig. 1:**
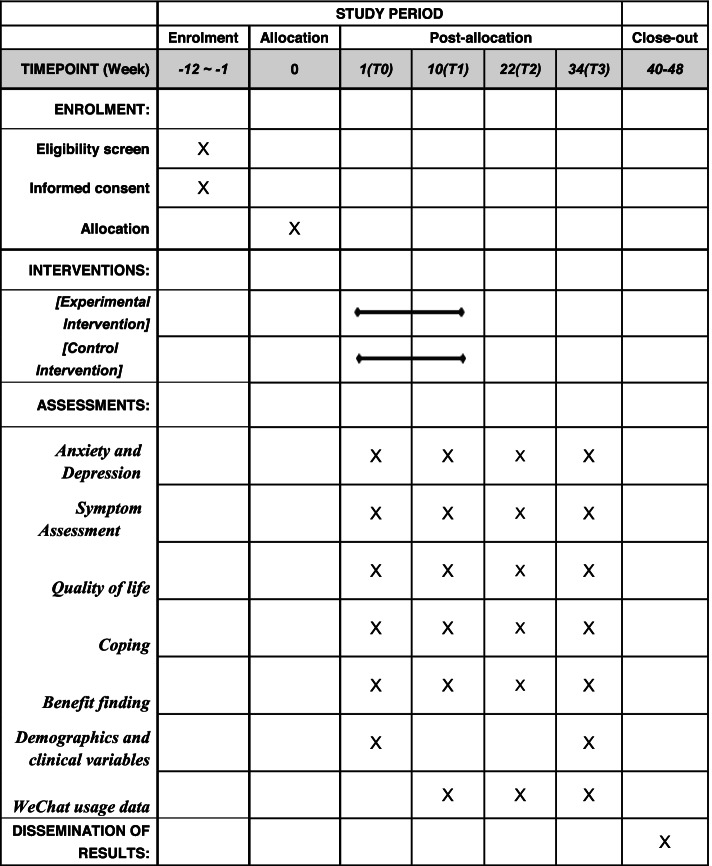
SPIRIT schedule of enrolment, interventions, and assessments

**Fig. 2:**
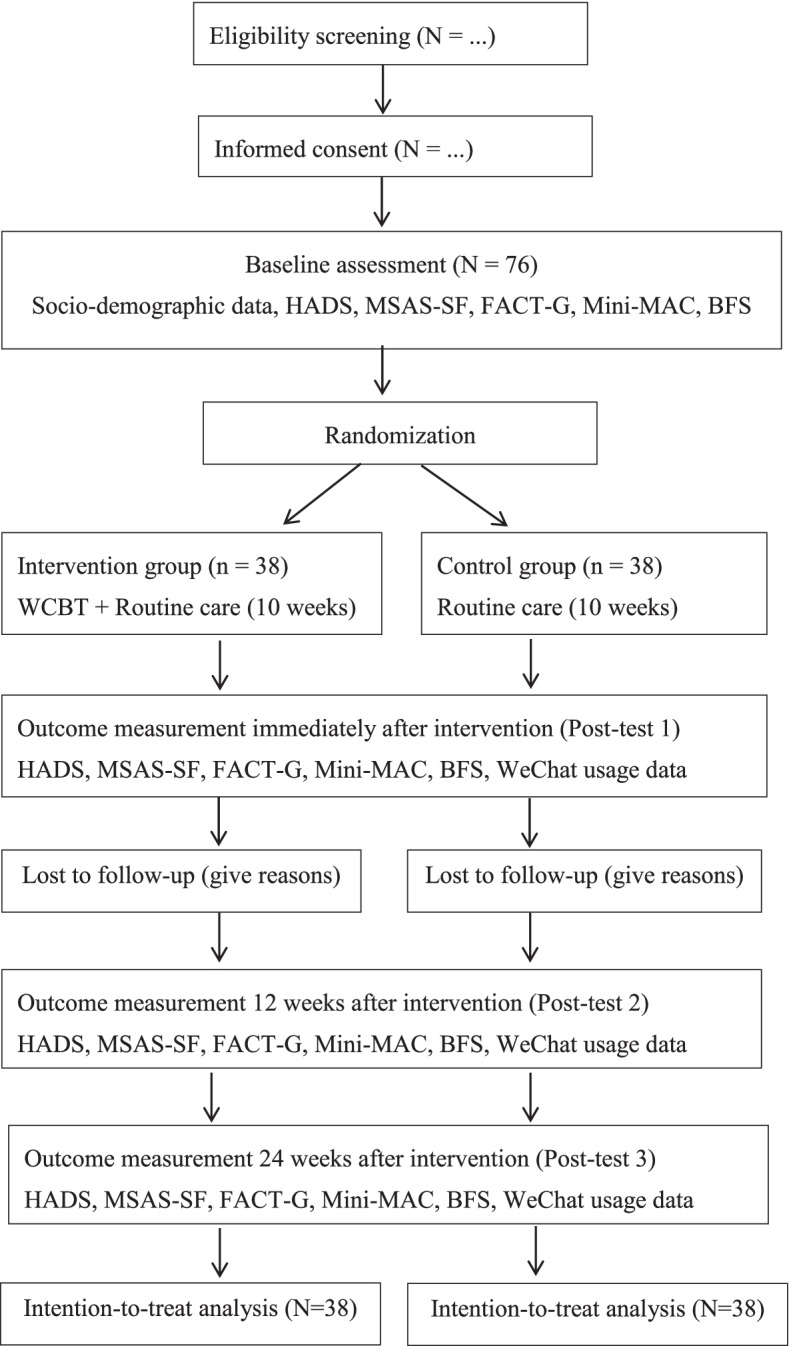
CONSORT flowchart of the study. Note: HADS: Hospital Anxiety and Depression Scale; FACT-G: Functional Assessment of Cancer Therapy—General; MSAS-SF: The Chinese version of the Memorial Symptom Assessment Scale- Short Form; Mini-MAC: Mini-Mental Adjustment to Cancer Scale; BFS: Benefit-Finding of Cancer Patients Scale

The study follows the SPIRIT 2013 Statement and the guidelines for the Standard Protocol of Clinical Trials [41]. Figure 1 is available for this protocol. The Consolidated Standards of Reporting Trials (CONSORT) [42] flowchart is presented in Fig. 2.

#### Study setting and participants

The study will be conducted in a tertiary public hospital affiliated to a university in China. Patients with gynecological malignant tumors (i.e., cervical cancer, endometrial cancer, and ovarian cancer) will be recruited in the outpatient department of the hospital.

##### Inclusion criteria

(1) patients with pathologically confirmed cervical cancer, endometrial cancer, and ovarian cancer; (2) patients who have completed surgery and / or radiotherapy and chemotherapy and have entered the rehabilitation stage; (3) Age 18–65 years; and (4) Kamofsky performance status (KPS) score > 60 points [43].

##### Exclusion criteria

(1) patients with history of mental illness, material dependence or consciousness disorder; (2) patients with hearing and visual impairment; and (3) patients undergoing any form of psychotherapy.

##### Drop-out criteria

(1) the disease progressed to a great extent during the intervention (tumor recurrence or metastasis); and (2) Other forms of psychotherapy are received during the study.

Sample size will be estimated based on previous research with a medium to small effect size [44]. With a power of 0.90, an alpha set at 0.05, and an effect size of 0.47 for the primary outcome of FACT-G scores, each group will be 29 women [21]. We estimate a 30% attrition rate based on an attrition rate of 28.8% in previous research [45]. A minimum of 76 women (38 in each group) are required.

#### Recruitment

The participants will be recruited from the gynecology outpatient department. Posters and flyers about this study will be posted and distributed in the outpatient center. An information sheet about the study will be given to the patients who verbally expressed their intention of participation. In order to make sure that they have enough time to read the information sheet, discuss with family members, and make the final decision, one week will be given to the patients. If a patient agrees, the contents, procedures, and rules of the study will be explained to her, and she will be asked to sign an informed consent. At the same time, oncology experts of this study will review the participants' medical records to obtain information about disease diagnosis, pathology, past and current cancer treatment, and patients' treatment compliance. Subjects will be screened according to the inclusion criteria. The recruitment period is expected to last for 3 months. Data will be collected before randomization (baseline), immediately after intervention (test 1), 3 months after intervention (test 2) and 6 months after intervention (test 3). The trial will be conducted in accordance with the Declaration of Helsinki and will take place from Jun 1, 2022 to Dec 31, 2023.

#### Randomization and blinding

Using the Excel software, the participants will be randomly divided into an intervention group and a control group with a ratio of 1:1. The randomization result will be placed in an opaque sealed envelope. Throughout the study, the participants, evaluators who will collect the data, and the data analysts will be blinded from the grouping information.

### Intervention

Patients in the control group will receive routine outpatient follow-up, during the visits, they will be provided general health recommendation, brief medication and exercise guidance, and information related to mental health. In addition, telephone follow-up will be carried out once a month. The patients are allowed to ask questions related to the disease through WeChat or mobile phone, yet they cannot participate in the online educational intervention.

For the intervention group, patients will receive group CBT for 10 weeks, carried out by psychiatrists, psychological counselors, and oncology nurses who had received CBT training. The details of intervention are described in Table 1. For the purpose of better communication, 38 patients in the intervention group were divided into three WeChat groups (12–13 patients in each group). The same intervention scheme was used in the three groups. Group CBT professionals will be invited to evaluate and supervise the intervention process.

**Table 1:** The details of intervention

	Intervention Group	Control Group
Service	WCBT + routine care	Routine care
Provider	Researchers	Doctors and nurses
Place	Web environment	Hospital outpatient
Form	WeChat platform	Face-to-face
Contents	Cognitive reconstruction; Cognitive behavioral therapy; Support expression	Regular follow-up
Duration and frequency	The total intervention time not less than 10 weeks	Follow up at least twice
Assessment	Baseline (pre-intervention) Test 1 (immediately after intervention) Test 2 (3 months after intervention) Test 3 (6 months after intervention)	Baseline (pre-intervention) Test 1 (immediately after intervention) Test 2 (3 months after intervention) Test 3 (6 months after intervention)

The contents of CBT intervention is based on currently mature cognitive and behavioral treatment programs in the world [46–48], which includes psychological education and self-monitoring, cognitive reconstruction, behavior correction, relaxation training, and problem-solving technique. The contents can be divided into three components: (1) cognitive reconstruction: the purpose is to explain disease- and psychology-related knowledge for patients at different stages, such as the risk factors, common treatment methods, prognosis, common side effects and treatments, and common psychological problems of gynecological malignant tumors; (2) cognitive behavioral therapy, the purpose of which is to identify and change incorrect psychological cognition, and train the patient’s coping skills. It will be used through the treatment plan of this study. The core is to help patients correctly manage stress and improve self-confidence, identify and optimize the use of social support resources, etc.; (3) support expression. A topic will be selected for WeChat group discussion. Members share their experiences and emotional exchanges about the topic, and the members will be encouraged to talk about stress and negative emotions, so as to help members support each other and reduce loneliness. There will be 6 topics throughout the study: (a) start treatment with your medical team. Including self-introduction and mutual understanding; joint formulation of group norms; sharing of the illness experience and current troubles; expressing the feelings and hopes of participating in the group; (b) management of symptoms and side effects, such as fatigue, pain, and insomnia. In addition, how to identify physical tension and use relaxation techniques (meditation, muscle relaxation, etc.); (c) the role of emotion during rehabilitation. How to identify negative thoughts and share them; (d) body image and sex; (e) family and friends; and(f) self-management of a healthy lifestyle.

First, online education information, in the format of texts, pictures, sound and video will be shared twice a week through WeChat. Before or after the release of information, the patients will be reminded via WeChat notifications. The learning time per week should be no less than 1 h. All previous information will be available for the participants at any time throughout the study. Secondly, the participants are required to carefully study the educational content, and turn in homework to a researcher. Homework includes learning experience, self-reflection diary, self-portrait videos of various relaxation exercises, etc., the purpose of homework is to improve the self-learning and monitoring ability of the participants, and then the participants are expected to extend the methods to their daily life, thereby strengthening the effect of intervention. In addition, discussions in the WeChat group will be held every 1–2 weeks based on the educational content released weekly. A gift will be given to the participants who actively participate in the discussion. Throughout the intervention, the participants will be encouraged to raise questions and share their experiences at any time.

### Outcome measurements

#### Primary outcomes

Hospital Anxiety and Depression Scale (HADS) [46]. There are 14 items, including 7 items on anxiety and 7 items on depression. A score of 0–7 means the participant is asymptomatic, 8–10 is suspicious, and 11–21 means symptomatic. Cronbach α coefficients of the total scale, as well as the anxiety and depression subscales are 0.88, 0.81 and 0.81, respectively [49].

Memorial Symptom Assessment Scale–Short Form (MSAS-SF), which is used to evaluate the characteristics of physical and psychological symptoms of cancer patients [50]. There are 32 items consisting of 2 subscales: physical symptoms and psychological symptoms. The incidence and frequency of each symptom will be evaluated. The Cronbach's alpha coefficient ranged from 0.84 to 0.91 [51].

#### Secondary outcomes

Functional Assessment of Cancer Therapy–General (FACT-G). It is a general scale used to evaluate the cancer patients' quality of life. There are 27-item consisting of 4 subscales: physical, social, emotional and functional wellbeing (total score of 0–108; higher scores = better QoL), Cronbach α is 0. 88, 0. 82, 0. 87, 0. 83,respectively [52].

Coping, which is measured with the mini-Mental Adjustment to Cancer Scale (mini-MAC) [53], a 29-item scale yielding 5 factors: Fighting Spirit, Helplessness/Hopelessness, Anxious Preoccupation, Fatalism, and Cognitive Avoidance. Items are responded to on a 4-point scale ranging from ‘definitely does not apply’ to ‘definitely does apply’.

Benefit Finding of Cancer Patients Scale(BFS). The scale was adapted by Weaver et al. [54], which was employed in prior studies of gynecological cancer patients [55]. Benefits included both interpersonal and intrapersonal changes from cancer. Higher scores indicated perceiving more benefits from cancer.

#### Other outcomes

Demographics and clinical variables. The information will be collected for each patient, including age, sex, marital status, educational level, occupation, work status, level of income, diagnosis, stage of disease, and treatment type.

WeChat usage data, which is used to evaluate the usage of WeChat official account by the participant, such as the frequency of login, browsing time.

### Data collection

The patients will be identifed by a unique study inclusion number and by the frst initial of their surname and of their given name. Baseline data will be collected face-to-face immediately after the patients signing the informed consent. Test 1 will be performed immediately after the intervention, test 2 and test 3 will be performed 3 months and 6 months after the intervention, respectively. The "questionnaire star" app on the WeChat platform will be used during test 1, test 2 and test 3 to send online electronic questionnaire to each participant. The questionnaires included HADS, FACT-G, MSAS-SF, mini-MAC, BFS, socio-demographics, and clinical data.

### Statistical analysis

All data were analyzed using the Statistical Package for Social Sciences 22.0(SPSS Inc., Chicago, IL, USA). Two researchers will carry out data analyses.Participants’ characteristics and outcome scores were summarized using means ± standard deviation (SD) or medians [interquartile ranges (IQR)] for continuous variables or frequencies (percentages) for categorical variables. Baseline characteristics and outcomes were compared using independent samples t-test or Mann–Whitney U test from continuous variables and chi-squared test for categorical variables between the intervention and control group. *p* values < 0.05 were considered statistically significant. All analyses will be conducted on an intention-to-treat basis.

### Validity and reliability

The feasibility of this study design is high, and the measurement tools have high effectiveness and reliability. The statistical analysis method is appropriate, which can effectively reduce error. In addition, because the evaluators and data analysts are blinded from the patient group, biases in the evaluation of intervention outcome can be effectively controlled.

## Discussion

Due to the lack of human and material resources in China, cancer patients are often not provided with effective interventions to ensure their physical and mental health. Under China's current medical and health system, it is more and more imperative to develop a time-saving, efficient, and accessible psychological intervention model that is suitable for local communities. The present study will be the first RCT on the use of CBT through WeChat on Chinese patients with gynecological malignant tumors in the rehabilitation stage. This study can provide evidences for the effectiveness of WeChat on supporting the rehabilitation of patients with gynecological malignant tumors.

### Limitations

In this study, a randomized controlled trial is design to study the effect of intervention. There are some limitations: (1) the sample size of the study is small, and due to limited resources, all participants are from level-3 hospitals, which may compromise the applicability of the study results. (2) Only patients with gynecological malignant tumors are included in this study, and the results of such intervention in other cancer populations needs to be verified. (3) the participants are limited to those who are willing to participate in this study through the WeChat platform, and the results cannot be extended to those who refuse to or cannot use WeChat.

## Data Availability

Not applicable.

## Abbreviations

IARC: International Agency for Research on Cancer
ICBT: Internet cognitive behavioral therapy
WCBT: WeChat-Based Cognitive Behavioral Therapy
HADS: Hospital Anxiety and Depression Scale
FACT-G: Functional Assessment of Cancer Therapy—General
MSAS-SF: The Chinese version of the Memorial Symptom Assessment Scale- Short Form
Mini-MAC: Mini-Mental Adjustment to Cancer Scale
BFS: Benefit-Finding of Cancer Patients Scale
